# Precision Lifetime Measurements Using the Recoil Distance Method

**DOI:** 10.6028/jres.105.007

**Published:** 2000-02-01

**Authors:** R. Krücken

**Affiliations:** A. W. Wright Nuclear Structure Laboratory, Physics Department, Yale University, New Haven, CT 06520; Nuclear Science Division, E. O. Berkeley National Laboratory, Berkeley, CA 94720

**Keywords:** differential decay-curve method, Gammasphere, groundstate deformation, magnetic rotation, New Yale Plunger Device, nuclear level lifetimes, nuclear structure, recoil distance method, shears bands, superdeformation

## Abstract

The recoil distance method (RDM) for the measurements of lifetimes of excited nuclear levels in the range from about 1 ps to 1000 ps is reviewed. The New Yale Plunger Device for RDM experiments is introduced and the Differential Decay Curve Method for their analysis is reviewed. Results from recent RDM experiments on SD bands in the mass-190 region, shears bands in the neutron deficient lead isotopes, and ground state bands in the mass-130 region are presented. Perspectives for the use of RDM measurements in the study of neutron-rich nuclei are discussed.

## 1. Introduction

The knowledge of matrix elements for transitions between excited nuclear levels adds important insight into the structure of nuclei. Relative transition matrix elements can be determined by measuring gamma-ray branching ratios but absolute transition matrix elements are only accessible by measuring the lifetime or the Coulomb excitation cross sections of the nuclear levels. Thus the knowledge of lifetimes of excited nuclear states is an important addition to the information available from other spectroscopic tools such as the measurements of γ-ray energies, angular distributions and polarizations. The reduced transition probability *B*(E2) for the $2_{1}^{+}\to 0_{1}^{+}$ ground state transition, for example, provides a measure of the charge quadrupole moment and thus the deformation of the ground state of an even-even nucleus. Other examples for the importance of transition matrix elements include the possibility to identify collective excitation modes on the basis of the magnitude and spin dependence of *B*(E2) values, or their sensitivity to the mixing of different configurations, in particular in the case of shape coexistence.

The precision achievable in lifetime experiments has dramatically increased with the availability of large, highly efficient γ-ray multi-detector arrays [1]. While this is due to the enhanced γ-ray detection efficiency it is also the use of γ-γ and higher fold coincidences that has improved the precision of lifetime measurements significantly. These instruments have also enabled us to access exotic, weakly populated nuclear excitations for lifetime measurements, such as superdeformed (SD) bands, which in some mass regions are only populated with a few percent of the total fusion cross-section. In particular for such exotic nuclear excitations lifetime measurements have proven to be essential for a sound understanding of the excitation mechanism. The most striking example for this is given by the shears bands in the Pb region [2,3], where the lifetimes were uniquely capable of showing the validity of the new concept of magnetic rotation [4].

This contribution will review some recent achieve ments that have been made in measuring nuclear level lifetimes in the picosecond range, which is a critical lifetime range for low-lying collective excitations, as well as some levels in more exotic nuclear excitations. The most commonly used method is the so-called recoil distance method (RDM) [5], also called the recoil distance Doppler-shift (RDDS) technique. Both terms are used interchangeably in the course of this article.

After pointing out some recent developments in the techniques for experiment and analysis in Sec. 2, results from several recent RDM measurements are presented in Sec. 3, which highlight the progress made in this field. Finally, Sec. 4 contains some perspectives for the use of RDM experiments in studying neutron rich nuclei using stable beams as well as radioactive ion-beams (RIBs).

## 2. Technical Developments for RDM Experiments and Their Analysis

The RDDS technique is the standard method to measure lifetimes of excited nuclear states in the picosecond range. The method uses a target chamber, called a plunger device, that contains a stretched target-and stopper-foil which are mounted parallel to each other at a variable distance. Excited states in the nucleus of interest are populated^1^ in the target foil. The nucleus then recoils with a velocity of a few percent the speed of light in the direction of the stopper foil where it is stopped. The distances are chosen such that the flight time is on the order of the effective lifetime^2^ of the levels of interest and vary typically from about 10 μm to several millimeters. In some cases the second foil is chosen to slow the recoiling nucleus down without stopping it but the basic principle of the method stays the same. The lifetime of a level of interest is determined from the changing intensities of fully Dopplershifted and stopped (or slowed) γ-ray components detected by the surrounding γ-ray detectors when varying the target-to-stopper distance. While the basic concept of the method is straightforward, the details of the techniques for experiment and analysis are fairly sophisticated. Here only a couple of points will be pointed out without going to deeply into the details, which can be found in the references.

### 2.1 The New Yale Plunger Device (N.Y.P.D.)

Recent years have witnessed significant developments of the plunger devices used for the RDM experiments. A modern plunger device needs to fit into the centre of a large multi-detector γ-ray array to take advantage of the high efficiency of such a system. At the same time the amount of material in the vicinity of the target has to be minimized so that the emitted γ rays can reach the surrounding detectors without being absorbed or scattered. At the same time it remains essential that the targets are flat and stretched so that they can be positioned accurately at very short distances from each other and remain flat under bombardment by the heavy-ion beam. This requires a stable, yet lean mechanical system to mount target and stopper foil with the ability to move the target with an accuracy of a few tenths of a micrometer. A measurement of the actual target-to-stopper distance is done by measuring the capacitance of the parallel-plate capacitor formed by target and stopper foil as described by Alexander and Bell [6]. The target-to-stopper distance is typically measured with respect to the electrical contact of the foils since the determination of an absolute distance is very difficult. Another technical requirement results from the fact that even in reactions with medium mass beams the thermal expansion of the mechanical mounts of target and stopper foil can be as large as a few micrometers. In γ-γ -coincidence measurements the bombarding time per distance is of the order of a few hours to about a day. During these time periods the beam current typically does not stay constant enough to keep the temperature of the mechanical mounts in thermal equilibrium. Therefore it is necessary to compensate the resulting changes of the target-to-stopper distance by some feedback mechanism that continually determines the target-to-stopper distance with beam on target and keeps it constant to a few percent of the absolute target-to-stopper distance.

A plunger device that fulfills all of the above requirements is the New Yale Plunger Device (N.Y.P.D.) which follows the design of the most recent Cologne plunger [7]. The foils remain parallel at all distances by moving the target with a system of concentric tubes that prevents tilting. A piezoelectric motor is used to move the target to a position of choice. Once in position the distance is kept constant by a feedback system that measures the capacitance and makes corrections of the distance by using a piezoelectric crystal that is mounted forward of the motor. With its continuous dynamic range of ±10 μm it is possible to compensate changes in the distance of less than 0.1 μm. Before beam is put on target the capacitance is calibrated with respect to a micrometer gauge head with a resolution of 0.02 μm in its most sensitive range. For the driving of the motor, the calibration of the capacitance and the feedback system a PC-based system controlled by a LabView™ program has been developed at Yale which also uses control electronics designed by H. Tiesler [8]. This system can be placed outside the target room at any desired distance, connected to the plunger device by only 10 BNC cables.

### 2.2 The Differential Decay Curve Method

Besides the developments in the design of plunger devices there has been a significant improvement of the reliability of lifetimes from RDM experiments due to the so called Differential Decay-Curve Method (DDCM) [9,10], which will be outlined briefly below.

If the level of interest is populated by transition *B* and de-populated by transition *A* a value τ(*x*) for the lifetime can be calculated at each target-to-stopper distance *x* by:

$$\tau \left(x\right)=\frac{F\left(x\right)}{G\left(x\right)}\equiv \frac{I_{u}^{A}\left(x\right)-\alpha \cdot I_{u}^{B}\left(x\right)}{\upsilon \cdot \frac{d}{dx}I_{s}^{A}\left(x\right)}$$ (1)

where

$$\alpha \equiv \frac{I_{u}^{A}\left(x\right)+I_{s}^{A}\left(x\right)}{I_{u}^{B}\left(x\right)+I_{s}^{B}\left(x\right)}.$$

**Fig. 1:**
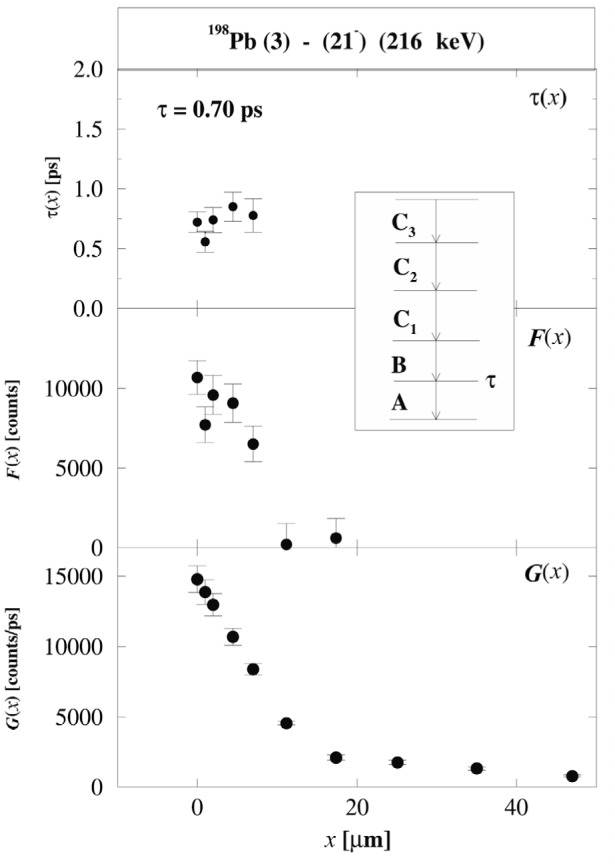
Values τ(*x*) for the level lifetime of the (21^−^) state in the shears band 3 of ^198^Pb as well as values for *F*(*x*) and *G*(*x*) from Eq. 1. It is apparent that *F*(*x*) and *G*(*x*) express the same dependence on the distance *x* and their ratio τ(*x*) is constant over the distance range where *F*(*x*) and *G*(*x*) are maximal.

Here $I_{u,s}^{A,B}$ are the un-shifted (u) and shifted (s) intensities for transitions *A* and *B*, respectively, in coincidence with the shifted component of any of the higher lying transitions *C*_i_ (see insert of Fig. 1). The gating conditions require coincidences with higher lying transitions, thus selecting nuclei with a particular decay path and eliminating any contributions of side-feeding in the lifetime analysis. This is an important advantage in the analysis of RDM data using γ-γ -coincidences as compared to the analysis of γ-singles data or gates below the level of interest. Figure 1 shows τ(*x*), *F*(*x*), and *G*(*x*) for the (21^−^) level in the shears band 3 of ^198^Pb. The τ-values are constant within the range of distances where *F*(*x*) and *G*(*x*) are large. This constancy of the τ(*x*)-curve provides a sensitive feedback for the analysis.

The DDCM differs substantially from the standard analysis of fitting linear combinations of exponential function. In the DDCM analysis a value for the lifetime of a given level is calculated directly from the observed coincidence intensities for each of the target-to-stopper distances. This makes the analysis independent of the analysis of higher lying states and of any feeding assumptions (if gates from above were used). The feedback provided by the constancy of the τ-values is an important tool to achieve reliable results since potential problems in the analysis can easily be spotted. The standard analysis, which is based on a χ^2^-fit of the decay curve, does not provide such a sensitive feedback since the χ^2^-minimization is only as good as the function used for the fit. Therefore erroneous parameters cannot be easily spotted, since in some cases a good fit of the decay curve might be obtained even with the wrong set of parameters.

Another benefit of the DDCM analysis in coincidence with feeding transitions is the fact that the deorientation effect, which influences the angular distributions for the low-spin states, is corrected automatically in the DDCM coincidence analysis, as was shown by P. Petkov [11].

In summary, the use of the DDCM for the analysis of γ-γ-coincidence data has improved the reliability of the results significantly since various systematic uncertainties such as feeding and side-feeding times and the deorientation effect do not affect the analysis, which also has a consistency check built in.

## 3. Recent Results From RDM Measurements

The resolving power of modern γ-ray multi-detector arrays has enabled the application of the RDDS technique to excited levels that are populated only with a few percent of the total fusion cross section. At the same time the precision for lifetimes in strongly populated structures, such as states on the YRAST line, has improved dramatically. In this section a few recent cases are reviewed as examples where RDM experiments have had considerable impact on the understanding of the underlying structure of the nuclei under investigation.

### 3.1 The Decay out of Superdeformed Bands in the Mass-190 Region

The sudden disappearance of the intensity at the bottom of superdeformed (SD) bands has been a puzzling and intensively investigated problem. Only recently, major breakthroughs have been accomplished with the first observations of discrete linking transitions between the SD bands in ^194^Hg [12,13] and ^194^Pb [14–16] and the respective normal deformed (ND) levels. These observations have for the first time enabled the determination of the excitation energies, spins and, in the case of ^194^Pb, parity [16] of superdeformed states in the 190-mass region. It was also possible to measure lifetimes at the bottom of some SD bands in the mass-190 region using the RDDS technique [17–19]. For example, lifetimes of 2.6(7) ps, 5.5 (10) ps, 8.3 (17) ps, and 20.0 (69) ps [18] were extracted for the 14^+^, 12^+^, 10^+^, and 8^+^ levels in the YRAST SD band of ^194^Pb, respectively. The mechanism leading to the decay out of the SD bands can be understood in a simple mixing picture [19], where the lowest observed SD states mix with their nearest neighbouring normal deformed (ND) states with the same spin and parity. The transition quadrupole moments of the transitions in the SD bands were extracted in each of the RDM measurements and did not show a significant reduction compared to the SD quadrupole moments at higher spin. This simple finding was the first experimental indication that the mixing between SD and ND states has to be very weak. A further analysis using a statistical model to estimate the level density and transition probabilities of the highly excited ND states revealed that the squared mixing amplitude of ND states mixed into the SD states is less then 4 % for all SD states in the mass-190 region with a significant branch to ND states at lower excitation energy [17–19].

**Fig. 2:**
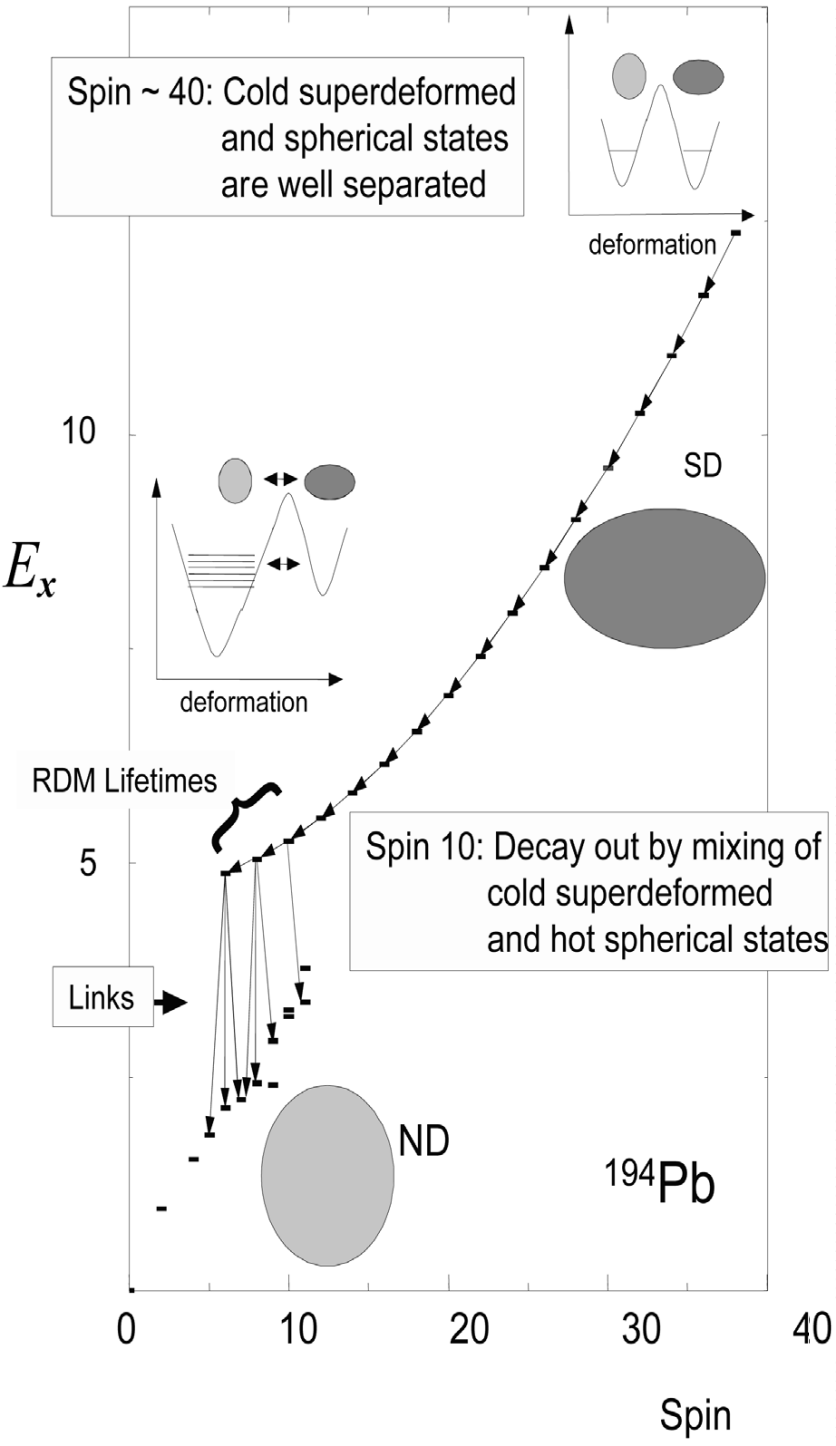
Energy vs spin plot showing the near-YRAST ND states and the YRAST SD states in ^194^Pb. The SD transitions as well as the observed linking transitions between SD and ND states are shown. The inserts schematically depict the potential energy vs deformation at the population region of the SD bands at high spins and the decayout region at low spins. In the population region both wells are of the same depth making the SD states YRAST or near YRAST states that can easily be populated in fusion evaporation reactions. The potential barrier between the wells separates the SD and ND states so that no mixing occurs for the lowest levels in the well. At low spins the SD well is shallow and at higher excitation energy with respect to the ND well. Thus the cold SD states are at the same level as hot, highly excited ND states with which they mix. The electromagnetic properties of these ND states determine the decay down to near YRAST states.

In the case of ^194^Pb many direct transitions between the lowest members of the SD band and the near YRAST states were observed [16] (see Fig. 2) and multipolarities of pure E1 and mixed E2/M1 character were determined. It is interesting to note that no stretched E2 transitions where observed. *B*(E1) values of about 8×10^−6^ W.u. and 5×10^−4^ W.u., respectively,^3^ were determined for the pure ND states at the excitation energy of the SD states, which are consistent with a statistical decay [18]. This implies that the ND states mixing with the SD states are highly mixed and contain only small amounts of wave-functions that are very similar to the structure of the SD states.

### 3.2 Shears Bands in the Pb Region

Very regular rotational level sequences connected by strong magnetic dipole (M1) transitions in the neutron deficient Pb isotopes[2,3] have been observed in recent years. The band head of these M1 bands is generated by the coupling of *h*_9/2_ and *i*_13/2_ protons to *i*_13/2_ neutron holes. These long quasi-particle spin-vectors ***j***_π_ and ***j***_υ_ are coupled almost perpendicular to each other, leading to a large perpendicular component ***μ***_⊥_ of the magnetic moment with respect to the total angular momentum vector ***J***. The orientation of this quantum system arises from an intrinsic anisotropy of the nuclear current density. In the case of the above described particle-hole coupling the observed rotational level sequence is governed by the rotation of the magnetic moment ***μ***_⊥_ around the total angular momentum vector. This leads to enhanced M1 transitions between the rotational levels and has given this phenomenon the name “magnetic rotation” [4].

Spin is generated in these M1 bands by the gradual alignment of the particle and hole angular momenta ***j***_π_ and ***j***_υ_ with the total angular momentum vector ***J***. This is reminiscent to the closing of the blades of a pair of shears, leading to the name “shears mechanism” [3]. This behaviour arises naturally in the framework of the tilted-axis-cranking (TAC) model [20]. The closing of the particle and hole spin-vectors with respect to the total angular momentum vector leads to a specific large drop of *B*(M1) values with increasing spin, since|***μ***_⊥_| is decreasing with decreasing opening angle and *B*(M1)∞|***μ***_⊥_|^2^ [20].

**Fig. 3:**
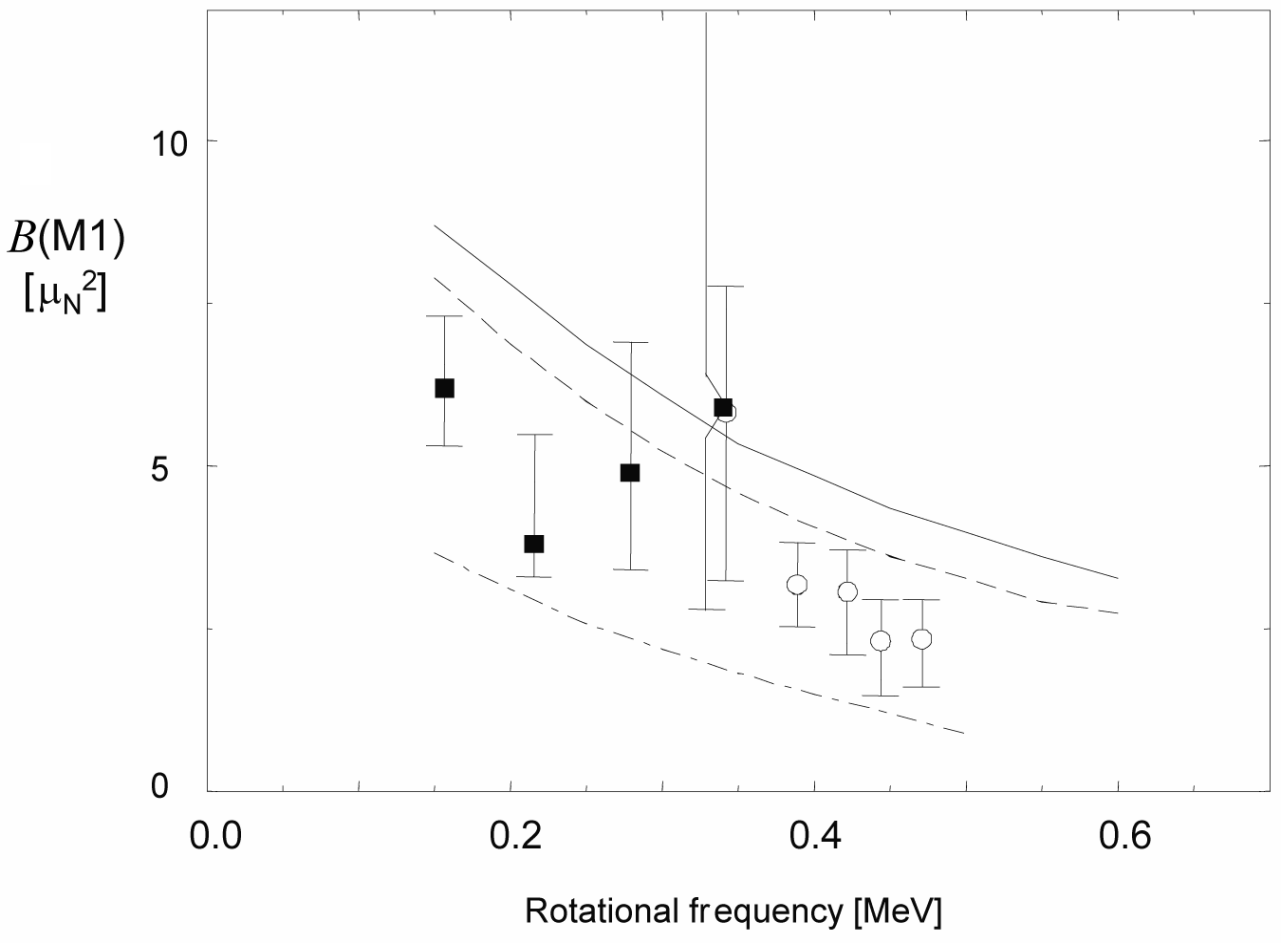
*B*(M1) values for the shears band 3 in ^198^Pb from a DSAM measurement [21] (open circles) and a recent RDM experiment [22] (filled squares). The various lines indicate predictions by TAC calculation for different possible configurations of this shears band. The agreement between experiment and theory provides strong support for the concept of magnetic rotation.

Many of the features of the shears bands have been described by the shears mechanism as well as models that did not involve this new concept. Lifetime experiments were carried out at Gammasphere [24] using the DSAM technique and RDDS technique in 193–199Pb [21,23] and ^198^Pb [22], respectively. Figure 1, for example, shows the DDCM analysis for the (21^−^) level in shears band 3 of ^198^Pb. The lifetime experiments have established the characteristic decrease of the *B*(M1) values with increasing spin as shown for shears band 3 in ^198^Pb in Fig. 3. These key observations have provided the essential support for the concept of magnetic rotation as the underlying mechanism for the shears bands. This is a very impressive example for the importance of lifetime measurements.

### 3.3 Lifetimes in the Ground Bands of Mass-130 Nuclei

The lifetime of the first excited 2^+^ level of an even-even nucleus is a measure for the deformation of the nucleus in its ground state. Thus the ratio of the transition quadrupole moments for transitions between states in the ground band and that of the 2^+^ → 0^+^ ground state transition is an excellent measure for possible changes in the deformation within the ground band. However, small changes in the deformation can only be detected if the lifetimes are measured with very high precision. Such accurate measurements have recently been performed for the first time using large γ-ray detector arrays.

Table 1 shows the relative transition quadrupole moments within the ground state bands of several Xe, Ba, and Nd nuclei [25]. The quadrupole moments are divided by the quadrupole moment for the 2^+^ → 0^+^ ground state transition. The lifetimes were measured with the GASP spectrometer of the INFN Laboratory Nazionali di Legnaro, Italy using the Cologne plunger and the DDCM for the analysis. The results in ^126^Ba are most impressive since, for example, the lifetime of the 4^+^ level was measured to be 8.59(18) ps [26] which represents a relative uncertainty of only 2 %. The ratios of the *Q*_t_ values are remarkably constant for most levels in the ground state bands below the crossing of the π(*h*_11/2_) or υ(*h*_11/2_) intruder bands. An exception is the band in ^126^Ba that shows clear deviations for the 6^+^ and 8^+^ levels, which are attributed to the underlying shell structure [26].

One can extract some very interesting information about the nuclear potential from the ratios given in Table 1, besides the general conclusion that a collective model interpretation is adequate for the ground state bands of the other nuclei in the table. Jolos et al. [27] have shown that, under rather general and reasonable approximations, the ratio

$$R_{4}=\frac{Q_{t}\left(4^{+}\to 2^{+}\right)}{Q_{t}\left(2^{+}\to 0^{+}\right)}\approx \frac{<0|{\left(Q\cdot Q\right)}^{2}|0{>}^{1/2}}{<0|Q\cdot Q|0>}=\frac{<\beta^{4}{>}^{1/2}}{<\beta^{2}>}$$

gives a direct measure of the stiffness of the nuclear potential with respect to the β-deformation. The nuclei in Table 1 are all very stiff with respect to the β-deformation, whereas they are known to be very soft with respect to the γ-deformation.

The example of ^126^Ba demonstrates that high precision lifetime measurements can help to investigate nuclear structure beyond the standard approach of simple collective models. More such measurements need to be performed in order to carry out critical tests of these models.

## 4. Perspectives

The examples given in the previous section have highlighted that important insights can be gained by the study of absolute nuclear transition matrix elements. Since major thrusts of current and future nuclear structure research are aiming at the study of nuclei far from stability, it is useful to take a look at the prospective areas in which the measurement of nuclear level lifetimes can make an impact in accordance with these goals.

### 4.1 Neutron-Rich Nuclei in the Heavy Rare Earth Region

**Fig. 4:**
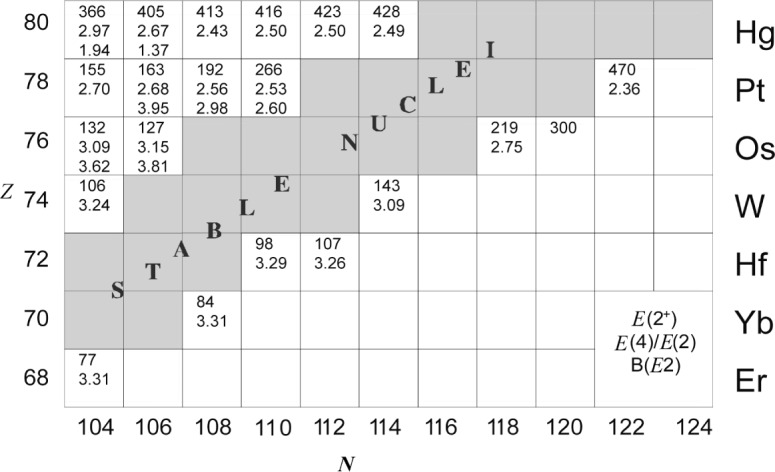
Nuclear chart for even-even nuclei in the neutron-rich *A* ≈ 100 region indicating the lifetime information currently available and the accessibility of nuclei in this mass region by different fission processes.

In regions of the nuclear chart that are difficult to access with experimental techniques the evolution of nuclear structure can often only be investigated by using a few key observables. The most basic observables available in even-even nuclei are the energy of the first excited 2^+^ state, the ratio *R*_4/2_ = E(4^+^)/E(2^+^) of the energies of the 4^+^ and 2^+^ states, and the reduced transition probability $B(E2;\;2_{1}^{+}\to 0_{1}^{+})$. The importance of these observables has been pointed out by Casten and Zamfir [28,29]. Figure 4 shows a part of the nuclear chart for even-even nuclei in the heavy rare earth region that indicates the measured values of these observables. It is remarkable that on the neutron-rich side very little information is available for nuclei only two and four neutrons away from the line of stability. These neutron-rich nuclei are too heavy to be produced as fission products, another major way of performing spectroscopy of neutron rich nuclei. However, these nuclei may be accessible via multi-nucleon transfer in deep inelastic collisions.

For some of these neutron-rich nuclei the energy of the first excited states is already known and it is the next task to measure the lifetime of the first excited 2^+^ level. This should in principle be possible by combining a plunger apparatus, such as the N.Y.P.D., with a position sensitive heavy-ion detector, such as the Rochester detector CHICO. In such an experiment neutron rich nuclei would be produced in deep inelastic collisions. Target-like and projectile like nuclei will fly through a retardation foil (instead of a stopper foil) in the plunger and will be detected in the heavy-ion counter. The nuclei can be identified and the kinematics of the reaction be reconstructed and thus RDM spectra for these nuclei can be produced and analyzed. This method certainly is in need of a highly efficient, very granular γ-ray detector array.

### 4.2 Neutron-Rich Nuclei in the Mass-100 Region

**Fig. 5:**
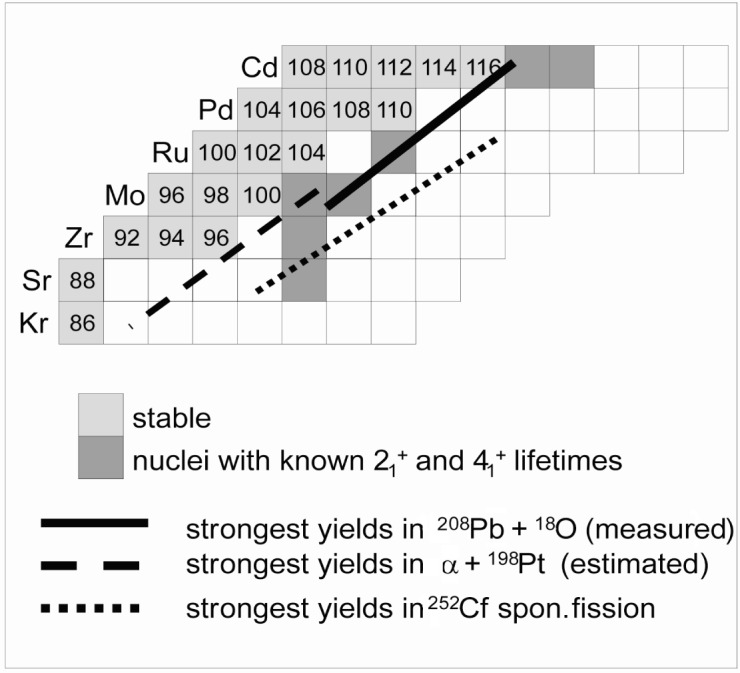
Nuclear chart for even-even nuclei in the neutron-rich *A* ≈ 100 region indicating the lifetime information currently available and the accessibility of nuclei in this mass region by different fission processes.

As already indicated a currently much utilized technique to study neutron-rich nuclei is the spectroscopy of prompt or β-delayed γ rays of fission fragments (see Ref. [31] for a recent review). This technique mostly uses spontaneous fission sources but also some spectroscopic information has been extracted from beam-induced fission. Lifetimes of low lying states have in the past mostly been measured using β-delayed γ rays and electronic time techniques but in a few cases the RDDS technique has also been employed [33]. Figure 5 shows a small portion of the nuclear chart for the even-even neutron rich Kr to Cd isotopes, indicating the lifetime information available for the first excited 2^+^ and 4^+^ levels. It is apparent that very little lifetime information is available in this mass region. At the same time the γ-ray spectroscopy of the nuclei in this region has indicated a wealth of structural phenomena. These include shape coexistence and shape transitions in the Sr and Zr nuclei, octupole correlations in the Mo isotopes, possible triaxiality in the Ru isotopes, and multi-phonon states in the Pd and Cd isotopes. With its transition from very deformed to nearly spherical shapes, this region provides an ideal testing ground for various collective models. Therefore the investigation of absolute transition matrix elements in this region is very important.

Figure 5 also schematically indicates the strongest yields for some examples of spontaneous and beam induced fission in this region. RDM experiments in this region are possible with the N.Y.P.D. in conjunction with the Yale Rochester Array for SpecTroscopy (YRAST Ball) [32] and an array of solar-cell detectors at backward angles. Such a solar-cell array has already been tested at Yale. One fission fragment will be detected in the solar-cells indicating that the other fragment went forward in the plunger. Thus γrays emitted by the forward flying fragments can be used for the RDM analysis. This technique was already used once by Mamane et al. [33] using only one Germanium detector and a thin ^252^Cf fission source. The use of a multi-detector system like YRAST Ball will help significantly in expanding the knowledge of nuclear level lifeitmes in this region.

### 4.3 RDM in Inverse Kinematics Coulex of RIBs

With the prospect of new radioactive beam facilites beams of exotic neutron rich beams will become available. These nuclei can be studied by Coulomb exciting them on a stable fixed target and from this type of data one can extract spectroscopic information about the level scheme as well as the transition and intrinsic matrix elements from the excitation cross sections. The reliability of the matrix elements is, however, sometimes dependent on the knowledge of all transitions going to and from a certain level as well as assumptions about the signs of the matrix elements involved. Here lifetime measurement with the RDDS technique could provide model independent absolute transition matrix elements. The technique works by Coulomb exciting the beam in a thin, low-*Z* target foil. The Coulomb excited beam nuclei are then slowed in a retardation foil. Here the beam nuclei can also be Coulomb excited. One can distinguish between the excitations in the two foils since γ rays emitted during the flight between the foils will be detected with a larger Doppler-shift than γ rays emitted after the retardation foil. As mentioned in Sec. 2.2, it is important to use gates on the maximum shifted components of higher lying transitions to obtain a reliable lifetime result for the levels of interest. This technique also will produce RDM spectra containing only γ rays from nuclei that were excited in the first foil. Such measurements need, besides the use of a state-of-the-art plunger, a highly efficient array of Ge-detectors such as Gammasphere or GRETA [34].

## Figures and Tables

**Table 1:** Relative transition quadrupole moments *Q*_t_ for several even-even *A* ≈ 130 nuclei

	$R_{4}=\frac{Q_{t}\left(4^{+}\to 2^{+}\right)}{Q_{t}\left(2^{+}\to 0^{+}\right)}$	$R_{6}=\frac{Q_{t}\left(6^{+}\to 4^{+}\right)}{Q_{t}\left(2^{+}\to 0^{+}\right)}$	$R_{8}=\frac{Q_{t}\left(8^{+}\to 6^{+}\right)}{Q_{t}\left(2^{+}\to 0^{+}\right)}$	$R_{10}=\frac{Q_{t}\left(10^{+}\to 8^{+}\right)}{Q_{t}\left(2^{+}\to 0^{+}\right)}$
^122^Xe	0.96 (2)	0.99 (4)		
^126^Xe	1.00 (3)	0.93 (6)	0.8 (1)	
^124^Ba	1.04 (2)	1.00 (3)	0.99 (6)	
^126^Ba	0.99 (2)	1.11 (3)	1.20 (5)	1.13 (7)
^128^Ba	1.00 (3)	0.98 (6)	0.9 (1)	0.7 (4)
^132^Nd	0.99 (2)	1.01 (2)		

## Footnotes

1 Typically a fusion evaporation reaction is used but also Coulomb excitation, transfer or the fission process can be used.

2 The effective lifetime describes the cumulative lifetime of a given level including all feeding times from cascades of levels above that level.

3 W.u. (Weisskopf unit) is defined as 6.45×10^−2^ *A*^2/3^ *e* ^2^ (fm)^2^ for E1 radiation, 5.94×10^−2^ *A*^4/3^ *e* ^2^ (fm)^4^ for E2 radiation, and 1.79 μ_N_^2^ for M1 radiation. Here *A* is the mass of the nucleus, *e* the electron charge, 1 fm (femtometer) equals 10^−15^ m, and μ_N_ is the nuclear magneton defined by *eħ/*2*m*, with *m* being the nucleon mass.
